# Integrating Gut Bacterial Diversity and Captive Husbandry to Optimize Vulture Conservation

**DOI:** 10.3389/fmicb.2020.01025

**Published:** 2020-05-25

**Authors:** Anne A. M. J. Becker, Stephen W. R. Harrison, Gerard Whitehouse-Tedd, Jane A. Budd, Katherine M. Whitehouse-Tedd

**Affiliations:** ^1^One Health Center for Zoonoses and Tropical Veterinary Medicine and Center for Conservation Medicine and Ecosystem Health, Department of Biomedical Sciences, Ross University School of Veterinary Medicine, Basseterre, Saint Kitts and Nevis; ^2^School of Animal, Rural and Environmental Sciences, Nottingham Trent University, Nottingham, United Kingdom; ^3^Kalba Bird of Prey Centre, Kalba, Sharjah, United Arab Emirates; ^4^Breeding Centre for Endangered Arabian Wildlife, Sharjah, United Arab Emirates

**Keywords:** gut microbiome, *ex situ* conservation, feeding ecology, husbandry, old world vultures, species recovery, prey diet

## Abstract

Endangered species recovery plans often include captive breeding and reintroduction, but success remains rare. Critical for effective recovery is an assessment of captivity-induced changes in adaptive traits of reintroduction candidates. The gut microbiota is one such trait and is particularly important for scavengers exposed to carcass microbiomes. We investigated husbandry-associated differences in the gut microbiota of two Old World vulture species using 16S RNA gene amplicon sequencing. Increased abundance of Actinobacteria occurred when vultures were fed quail but not rat or chicken. Conversely, diet preparation (sanitization) had no effect, although bacterial diversity differed significantly between vulture species, likely reflective of evolved feeding ecologies. Whilst the relative lack of influence of a sanitized diet is encouraging, changes in bacterial abundance associated with the type of prey occurred, representing a dietary influence on host–microbiome condition warranting consideration in *ex situ* species recovery plans. Incorporation of microbiome research in endangered species management, therefore, provides an opportunity to refine conservation practice.

## Introduction

For diverse reasons, many attempts to breed and subsequently reintroduce endangered species into their natural habitat from captivity have not been successful (Bowkett, 2009; Conde et al., 2013; Willoughby et al., 2015). One potential reason is the loss of adaptive traits (Araki et al., 2007; Willoughby et al., 2015), which are not only encoded by the host genetic architecture but also by the host-associated microbiome. The gut microbiome could be considered such an adaptive trait, representing a substantial community of microorganisms (and their collective genes) which play vital roles in host physiology (West et al., 2019) and potentially influences reintroduction success (Redford et al., 2012). In turn, the microbiome is under both genetic and environmental control, with diet acting as a pivotal determinant of gut microbial assembly (Spor et al., 2011). Over the past decade, knowledge of microbial symbionts in host health and disease has increased considerably. However, animal microbiome research has only recently been introduced as a perspective for modern conservation and species recovery practices (Redford et al., 2012; Chong et al., 2019; Trevelline et al., 2019; West et al., 2019).

Species recovery often necessitates movement of animals for translocation or captive breeding, but typically involves biosecurity protocols and anti-microbial prophylaxis (West et al., 2019), which are at odds with current appreciation for the symbiotic host–microbiome relationship. Hence, a paradigm shift is required to not only include microbial research as a fundamental component in species recovery programs, but to also consider co-extinction of host-associated microbes an undesirable outcome (Trevelline et al., 2019; West et al., 2019). In particular, the influence of husbandry factors on the gut microbiome of captive animals and consequently their health (and post-release survival) is poorly understood (Chong et al., 2019; Trevelline et al., 2019; West et al., 2019), notably in regard to specialized taxa.

Vultures are such specialists, well-known for their intimate interactions with pathogens. These obligate scavengers remove carcasses from the environment, and provide important ecosystem functions (Safford et al., 2019). Yet, vultures are now among the most threatened group of birds, suffering global population declines of >80% (Safford et al., 2019). Consequently, vultures have become the focus of intensive conservation efforts (Safford et al., 2019). Critical to vultures is their ability to safely consume carrion in varying stages of decomposition; an adaptation which is integrally linked to their gut microbiota (Roggenbuck et al., 2014). However, the gut microbiota of many vulture species remains largely uncharacterized with little known regarding the impact of consumption of sanitized food stuffs on the vulture microbiome in wild and captive settings.

## Materials and Methods

The aim of the current study was to investigate the potential impact of diet preparation on the specialized, luminal-bacterial alliance of two species of Old World vultures, the Griffon (*Gyps fulvus*) and Egyptian vulture (*Neophron percnopterus*). This was achieved by characterization of the luminal-microbiome using high-throughput amplicon sequencing of DNA form fecal samples collected after provision of diets prepared under divergent conditions. A secondary objective was identified *post hoc*, whereby prey type provisioning associated with fecal sample characterization permitted the *post hoc* investigation of the impact of prey type on luminal microbiota.

### Ethics Statement

This project was approved by the Nottingham Trent University’s School of Animal, Rural and Environmental Science Ethics Review Group (ARE76).

### Study Population, Experimental Design of Diets, and Sample Collection

Four Egyptian vultures (*Neophron percnopterus*) and 7 Griffon vultures (*Gyps fulvus*) housed at the Kalba Bird of Prey Centre (KBoPC) along with 4 Egyptian vultures housed at the Breeding Centre for Arabian Wildlife (BCEAW), both located in the United Arab Emirates (UAE), were used in this study (Table 1). To represent typical captive dietary provision (Gaengler and Clum, 2015), two dietary conditions were implemented in a semi-randomized cross-over study design. Birds were fed either a sanitized diet (SD) comprising an overall weekly mixture of dressed quail, chicken and rat carcasses [i.e., skinned, partially eviscerated (gastrointestinal tract removed)] which were washed under tap water, or an un-sanitized diet (UD) of fully feathered/furred, intact whole carcass of the same prey species. Daily rations comprised only single prey species, and the species consumed each day were recorded for the duration of the study. No intervention in terms of the choice of prey species offered per day was performed in order to best replicate normal husbandry conditions for captive vultures. Diets (sanitized or un-sanitized; see Supporting Information for further details) were fed for a period of 4 weeks with fecal sampling in the following (fifth) week. A 2-week washout period was then implemented, during which time the birds were fed a mixture of prey items prepared as per standard husbandry practices at each facility. This mixed diet included both dressed carcasses and intact prey items of the same species as fed during the study period. After the washout period, birds were fed the alternative diet for 4 weeks before fecal sample collection in the fifth week (with daily prey species consumed recorded as previously described).

**TABLE 1:** Vulture details, diet, and housing conditions at the time of study.

Species	Local ID	Sex	Age (years)	Origin	Phase 1 diet^a^	Phase 2 diet^a^	Facility^b^	Co-housed with	Aviary size and substrate	Genetic relationships
Egyptian vulture	EV002	M	6*	Wild, Oman	Clean	Dirty	KBoPC	EV005	Open air enclosure, 64 m^2^, natural rock and sand substrate	Unknown
Egyptian vulture	EV005	F	6*	Wild, Oman	Clean	Dirty	KBoPC	EV002	Open air enclosure, 64 m^2^, natural rock and sand substrate	Unknown
Egyptian vulture	EV001	M	6*	Wild, Oman	Dirty	Clean	BCEAW	EV003, EV004, EV006	Partially covered enclosure, 100 m^2^, natural sand substrate	Unknown
Egyptian vulture	EV003	M	6*	Wild, Oman	Dirty	Clean	BCEAW	EV001, EV004, EV006	Open air enclosure, 100 m^2^, natural rock and sand substrate	Unknown
Egyptian vulture	EV004	F	6*	Wild, Oman	Dirty	Clean	BCEAW	EV001, EV003, EV006	Open air enclosure, 100 m^2^, natural rock and sand substrate	Unknown
Egyptian vulture	EV006	F	6*	Wild, Oman	Dirty	Clean	BCEAW	EV001, EV03, EV004	Open air enclosure, 100 m^2^, natural rock and sand substrate	Unknown
Griffon vulture	GY003	F	15	Captive bred, UAE	Clean	Dirty	KBoPC	GY007, GY006	Open air enclosure, 1488 m^2^, natural rock and sand substrate	Parent to GY018 GY019
Griffon vulture	GY007	F	13	Captive bred, UAE	Clean	Dirty	KBoPC	GY003, GY006	Open air enclosure, 1488 m^2^, natural rock and sand substrate	Parent to GY015 GY016
Griffon vulture	GY006	M	14	Captive bred, UAE	Clean	Dirty	KBoPC	GY003, GY007	Open air enclosure, 1488 m^2^, natural rock and sand substrate	Parent to GY018 GY019
Griffon vulture	GY015	F	2.5	Captive bred, UAE	Dirty	Clean	KBoPC	GY016	Open air enclosure, 242 m^2^, natural rock and sand substrate	Offspring of GY005 GY003
Griffon vulture	GY016	M	1.5	Captive bred, UAE	Dirty	Clean	KBoPC	GY015	Open air enclosure, 242 m^2^, natural rock and sand substrate	Offspring of GY005 GY003
Griffon vulture	GY017	F	3.5	Captive bred, UAE	Clean	Dirty	KBoPC	None	Covered mews, natural sand substrate, wooden block with AstroTurf surface. Tethered and flown daily by falconry team	Offspring of Undetermined
Griffon vulture	GY018	M	0.75	Captive bred, UAE	Dirty	Clean	KBoPC	None	Covered mews, natural sand substrate, wooden block with AstroTurf surface. Tethered and flown daily by falconry team	Offspring of GY006 GY003
Griffon vulture	GY019	F	0.75	Captive bred, UAE	Dirty	Clean	KBoPC	None	Covered mews, natural sand substrate, wooden block with AstroTurf surface. Tethered and flown daily by falconry team	Offspring of GY006 GY003

^*a*^Diet comprised either a sanitized diet (SD) of dressed quail, chicken and rat carcasses (i.e. skinned, partially eviscerated (gastrointestinal tract removed)) which were washed under tap water, or an un-sanitized diet (UD) of fully feathered/furred, intact whole carcass of the same prey species. ^*b*^KBoPC = Kalba Bird of Prey Centre (UAE); BCEAW = Breeding Centre for Endangered Arabian Wildlife (UAE). *Estimated age (bird was not hatched in captivity).

Fresh fecal samples (approximately 2 g/bird) were collected by scraping or syringe suction from the surface (see Supporting Information). We collected multiple samples per bird during the sampling week on an opportunistic basis, i.e., when a bird was seen to defecate (therefore confirming ownership and freshness) and the fecal matter was accessible (i.e., having been voided onto a surface amenable for sampling) the sample was collected. All voidings meeting this sampling criteria were collected during the week of sampling. Samples were transferred into sterilized containers and then stored at −20°C for an average of 60 (maximum 114) days prior to transport to the laboratory (ABC Labs, Dubai, United Arab Emirates).

### DNA Extraction, Amplification, and Sequencing

Total bacterial community DNA extraction from each distinct fecal sample followed the conventional phenol–chloroform protocol (Pitcher et al., 1989). DNA size and integrity were assessed on 1% agarose electrophoresis gels. DNA extracts were then subject to Illumina MiSeq sequencing targeting the V4-16S rRNA gene region. The variable regions were amplified using a modified version (Apprill et al., 2015; Walters et al., 2015; Parada et al., 2016) of the original 515F-806R primer pair (Caporaso et al., 2011, 2012) and pooled libraries were constructed following the protocol as described by Kozich et al. (2013). Libraries were sequenced using 250 bp paired-end sequencing chemistry on an Illumina MiSeq platform as described previously (Kozich et al., 2013).

### 16S rRNA Sequence Read Processing

Pre-processing of sequencing data was done using scripts from the Microbiome Helper 16S Workflow (Comeau et al., 2017) and included stitching paired-end reads with PEAR (v0.9.10) (Zhang et al., 2014), quality assessment with FastQC (v0.11.5) (Andrews, 2010) and filtering based on read length and quality. The quality threshold score was set at 37 over at least 90% of the bases and reads shorter than 250 bp were removed. Following read filtering, potentially chimeric reads were screened out using VSEARCH (v1.11.1) (Rognes et al., 2016), which implements the UCHIME algorithm (Edgar et al., 2011). In this study, the filtered reads were classified into different operational taxonomic units (OTUs) following two approaches. First, we used an open-reference algorithm (Rideout et al., 2014) which clusters reads against a reference sequence collection (≥97% sequence similarity) and subsequently clusters sequences that do not match the sequence database *de novo*. The OTU table generated by this approach was used for all diversity and taxonomic analyses. The reference sequence collection used was the v.13_8 of the GreenGenes 16S rRNA gene database (DeSantis et al., 2006). OTUs having <0.1% of the total number of reads were filtered out and the OTU tables were rarefied to a minimal number of reads (11 150 seq).

### Statistical Analysis

#### Bacterial Composition According to Vulture Species and Diet Preparation

To assess sampling depth coverage and species heterogeneity in each sample, alpha diversity metrics were employed on rarefied OTU tables using observed species (i.e., total OTUs per sample) and Shannon’s diversity indexes. Beta-diversity was assessed by calculating unweighted and weighted UniFrac and Bray–Curtis distances (Lozupone et al., 2011), which were tested for significant differences between sample categories using non-parametric ANOSIM tests with 999 permutations on non-rarefied data. Relative abundances of OTUs at different taxonomic levels were assessed using non-parametric Kruskal–Wallis test with false discovery rate (FDR) correction for multiple testing. Our threshold for significance was *P* < 0.05. Analysis was done using scripts from QIIME (Caporaso et al., 2010), STAMP (Parks et al., 2014), and RStudio (RStudio Team, 2015). Differences in taxonomic relative abundance for each phylum between dietary conditions (UD vs. SD) and different prey types were tested using generalized linear models, with dietary conditions, prey type and vulture species as fixed effects, and individuals from different facilities as nested random effects. Likelihood tests were used for comparisons of the models to one another and to a null model that included only the nested random factor. Similarly, we tested for an effect of vulture species on alpha diversity measures (observed number of OTUs and Shannon diversity index) in the fecal samples by comparing a linear mixed-effects model that included vulture species, dietary condition and prey type to one that included only dietary condition and prey type. These analyses were carried out in the “lmer package” in R.

#### *Post hoc* Analysis According to Prey Type (Regardless of Diet Condition)

Effect of prey type appeared as an important variable during analysis described in 2.5.1. As such, records of prey consumed each day were subsequently matched to instances where a fecal sample had been produced and collected on the following day. This time lag was considered appropriate on the basis of a known ∼21 h mean digesta retention time determined in a separate study with this population of Griffon vultures (Daneel et al., 2019). Griffon vultures had fecal samples matched to a total of 18 quail-feeding days, and 12 rat-feeding days. Egyptian vultures had fecal samples matched to a total of 2 quail-feeding days, 12 chicken-feeding days, 5 rat-feeding days, and 3 fasting days. The effect of prey type was tested by modeling phylum abundance measures against prey type consumed the day prior to sample collection, regardless of vulture species or preparation condition of the diets. These analyses were carried out in the “lmer package” in R.

## Results

We collected 52 fecal samples from the 15 birds in our cross-over study design; each bird was sampled at least once per dietary condition (range 1–5 samples per condition), with an average of 4 samples per bird being collected.V4-16S rRNA gene sequencing and subsequent quality filtering generated 5,293,884 high-quality sequences, with an average of 101,805 reads per sample (minimum 11,150; maximum 867,136 reads per sample). Using a threshold of 97% identity, sequences clustered into 533 OTUs with an average of 236 ± 62 OTUs retrieved in Griffon vulture samples and 180 ± 77 OTUs in Egyptian vulture samples.

### Bacterial Composition According to Vulture Species and Diet Preparation

No significant impact of diet preparation (i.e., sanitization) was detected (*P* = 0.1454) for either vulture species. Nonetheless, patterns of change were detectable at the taxonomic family level in our birds whereby a general trend toward reduced abundance under sanitized dietary conditions was observed (Supplementary Figure 1).

**FIGURE 1:**
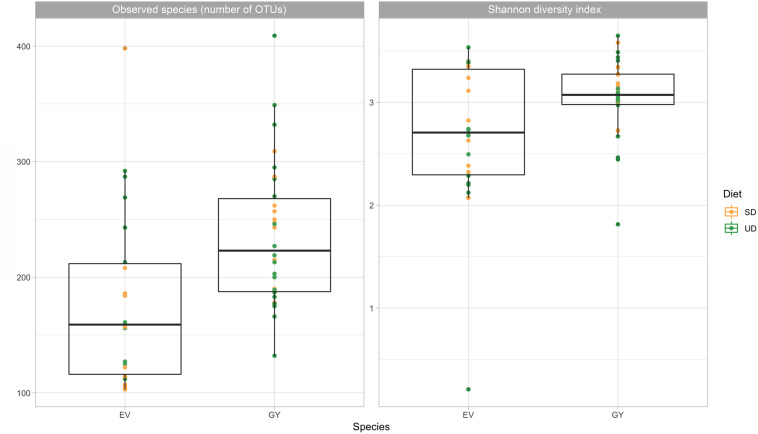
Variation in gut bacterial diversity between Egyptian and Griffon vultures. Alpha diversity based on rarefied data, measured by observed species and Shannon diversity Index, plotted for 52 fecal samples of two Old World vulture species (EV = Egyptian vulture, six individuals, *n* = 22 samples; GY = Griffon vulture, seven individuals, *n* = 30 samples). Statistical testing showed significant difference in observed species (Wilcoxon, *P* < 0.05) and Shannon diversity (Wilcoxon, *P* < 0.05) between both vulture species. Vultures were fed either a sanitized diet (SD) consisting of skinned, de-gutted and washed rats, chicken and quail, or un-sanitized diet (UD) consisting of intact whole rats, chicken and quail. No significant difference were observed between diets.

**FIGURE 2:**
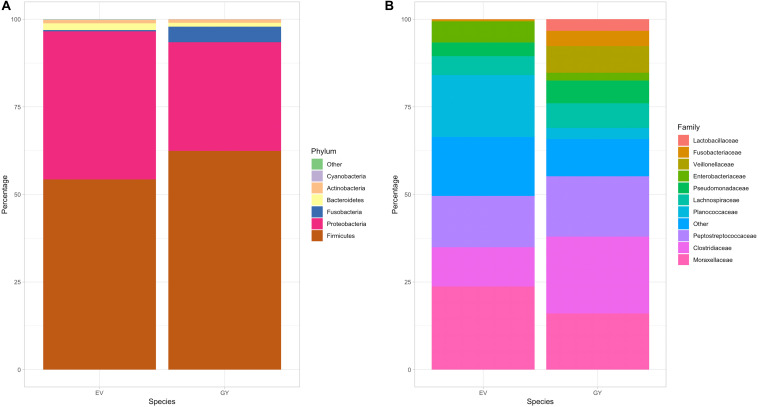
Gut bacterial composition of Egyptian and Griffon vultures. Taxonomic bacterial profile of 52 fecal samples from Egyptian (EV; six individuals, *n* = 22 samples) and Griffon vultures (GY; seven individuals, *n* = 30 samples) at phylum (**A**; left) and family (**B**; right) level. Of 75 families classified, only 14 with an abundance >1% of total reads are displayed.

Vulture species significantly affected fecal bacterial richness (*P* < 0.05) and Shannon diversity index was significantly different between vulture species (Figure 1; *P* < 0.01), but no overall effect of vulture species (*P* = 0.546) nor diet (*P* = 0.1454) or prey type (*P* = 0.2707) were observed in the full mixed-effects model. The gut bacterial community composition in both Griffon and Egyptian vultures was characterized by the dominance of genera within the phyla Firmicutes (58.4%) and Proteobacteria (36.6%) (Figure 2A). Within Firmicutes, sequences were classified into seven families with an abundance of >1% of total reads (Figure 2B). Clostridia dominated the bacterial community, represented by *Clostridiaceae* (17%) and *Peptostreptococcaceae* (16%). Fusobacteria (2.4%), Actinobacteria (1%) and Cyanobacteria (0.1%) were minor contributors to the vulture’s gut bacterial composition and Bacteroidetes represented 1.5% of the microbiome in the studied Griffon and Egyptian vultures.

**FIGURE 3:**
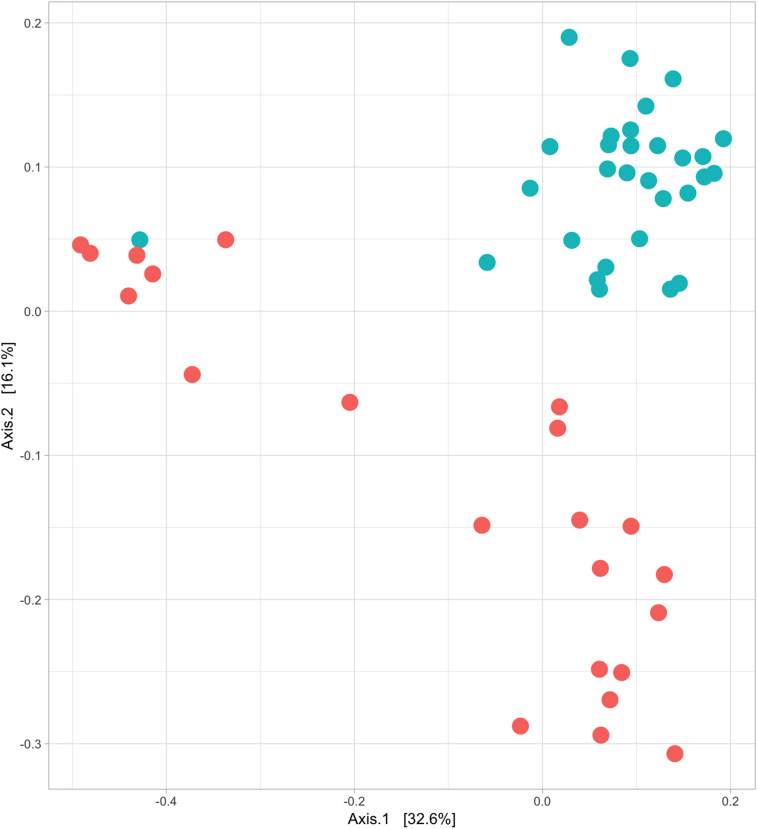
Egyptian and Griffon vultures exhibit different bacterial communities. Beta diversity; principal coordinate analysis visualizing the clustering of bacterial communities of 52 fecal samples from Egyptian (six individuals, *n* = 22 samples; red) and Griffon vultures (seven individuals, *n* = 30 samples; blue) based on unweighted UniFrac dissimilarity matrix. Vulture species exhibited minor overlap (ANOSIM; *R* = 0.545, *P* = 0.001).

Structural differences in bacterial community composition between species were also observed (Figure 3 and Supplementary Figures 2, 3). These differences were apparent at phylum level with a significantly higher relative abundance of Firmicutes (Welch’s *t*-test, *q* = 0.018) in Griffon vultures and of Proteobacteria (Welch’s *t*-test, *q* = 0.025) in Egyptian vultures (Supplementary Figure 4). Additionally, although not statistically significant, Fusobacteria were observed in a higher abundance and Bacteroidetes in lower abundance in Griffon vultures. No other metadata included in the mixed-effects models (age, location, aviary) had a significant impact on the gut bacterial diversity.

### *Post hoc* Analysis According to Prey Type (Regardless of Diet Condition)

**FIGURE 4:**
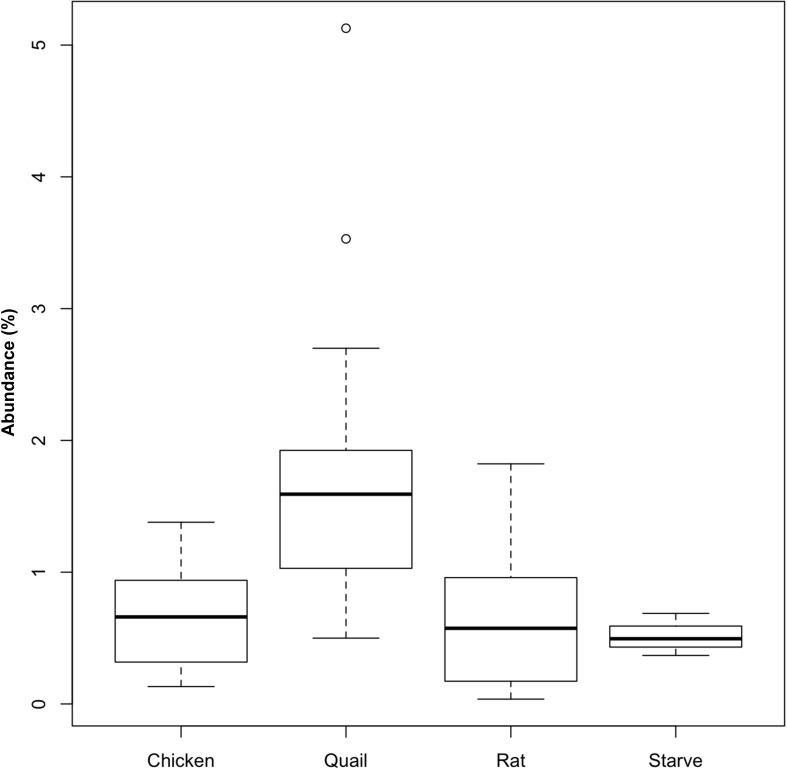
Relative abundance of Actinobacteria in the fecal bacterial community of vultures varied according to prey type. Boxplots showing the relative abundance of Actinobacteria in fecal samples from Griffon vultures (seven individuals, *n* = 30 samples) fed either rat (*n* = 12 samples) or quail (*n* = 18 samples), and Egyptian vultures (six individuals, *n* = 22 samples) fed either quail (*n* = 2 samples), rat (*n* = 5 samples), or chicken (*n* = 12 samples), or following a ‘fasted’ day (*n* = 3 samples). For quail and rat prey types, fecal Actinobacteria abundance data from both vulture species were combined, but differences between prey type were only statistically significant for Griffon vultures (*P* = 0.02). No statistical differences were detected between the four prey types fed to Egyptian vultures.

Griffon vultures exhibited a higher relative abundance of Actinobacteria (represented by 53 OTUs) when fed quail (*P* = 0.02; *n* = 18 samples) compared to when fed rats (*n* = 12 samples) (Figure 4). No equivalent effect of prey type was detectable for Egyptian vultures. The increase of Actinobacteria could be attributed to an increase in abundance of seven OTUs assigned to *Coriobacteriaceae* (Genus Rhodococcus, ∼21% of sequences assigned to Actinobacteria) and one OTU assigned to *Nocardiaceae* (∼ 24% of sequences assigned to Actinobacteria).

## Discussion

Our study represents the first ever empirical investigation of the hypothesis that captive dietary conditions could influence gut microbiota of an obligate scavenger (Blanco, 2014; Roggenbuck et al., 2014), with findings in support of a modifying role for prey type, but not diet preparation. In contrast to previously suggested links between feeding ground sanitization status and raptor gut microbiota (Gangoso et al., 2009; Blanco, 2014), no significant impact of diet preparation (sanitization) was detected. Rather, it appears that increased sanitization in zoos (Crissey et al., 2001), compared to free-ranging habitats, is unlikely to compromise vulture gut bacterial diversity. Nonetheless, the trend toward reduced bacterial abundance under sanitized dietary conditions aligns with the inoculation theory and warrants investigation utilizing larger, longitudinal studies.

Considering the bacterial composition observed, Bacteroidetes, typically a major phylum in many species including birds (Ley et al., 2008; Waite and Taylor, 2014), was only a minor contributor of the microbiome in our Griffon and Egyptian vultures. This is in accordance with the low proportions (<1%) of this phylum in three other Old World (Meng et al., 2017) and a New World vulture species (Rodrigues De Carvalho et al., 2003; Roggenbuck et al., 2014). Members of the Bacteroidetes are known to thrive on the plethora of complex polysaccharides that constitute “dietary fiber” (Thomas et al., 2011) and are correspondingly represented in lower proportions in species with higher dietary protein intake (Becker et al., 2014). Hence, this likely reflects vultures’ carnivorous nature and may explain their divergence from other (non-carnivorous) avian gut microbiomes. Inter-specific differences in bacterial composition detected in our study and others (Roggenbuck et al., 2014; Waite and Taylor, 2014; Meng et al., 2017) emphasize the need for caution in extrapolation of data between different vulture species, supporting recent calls to increase fundamental knowledge of animal microbiomes on a species-specific basis (Trevelline et al., 2019; West et al., 2019), including in conservation biology (Redford et al., 2012).

Diet specialization, along with phylogeny, is considered integral in shaping microbial diversity in a healthy vertebrate’s gut (Ley et al., 2008; Waite and Taylor, 2014). In the wild, Griffon vultures access the carcass directly during group feeding bouts to obtain protein- and fat-rich tissues, whereas the smaller Egyptian vultures rely on scraps of tissue picked up from the area surrounding the carcass (Kruuk, 1967; Hertel, 1994). Egyptian vultures also include insects in their diet, pick at bare bones, and have unusual coprophagic tendencies (Kruuk, 1967; Negro et al., 2002). This likely contributes toward a noteworthy fiber intake of plant (e.g., prey digestive tracts, feces) and animal (e.g., skin, bone, chitin, connective tissue) origin. This different feeding ecology could explain the lower proportions of (fat-adapted) Firmicutes and the relatively higher (fiber-adapted) Bacteroidetes detected in Egyptian vultures. A greater abundance of *Enterococcaceae* (associated with increased fiber intake and decreased *Lactobacillaceae*) [associated with decreased protein intake (Clarke et al., 2012)] in the Egyptian vulture could also reflect an evolved adaptation to these differences in feeding ecology. Likewise, fibrous prey components from the un-sanitized diets (e.g., skin, digestive tracts) may facilitate population growth of organisms associated with carbohydrate substrates such as *Bacteroidaceae* (Thomas et al., 2011) (observed here with a numerically higher abundance). Comparisons between free-ranging and captive birds using equivalent sampling and analyses techniques to avoid bias have not yet been conducted for Griffon and Egyptian vultures. Our findings serve as a valuable starting point for future comparative studies.

Unlike previous findings (Waite and Taylor, 2015), age, location, and aviary had no significant impact on the gut bacterial diversity. Importantly, data from co-housed birds did not cluster together and no clustering was apparent on the basis of housing location, despite multiple environmental differences (e.g., substrates, vegetation, aviary size, husbandry protocols, and neighboring species). Although similar to observations in New World vultures (Roggenbuck et al., 2014) and other avian species (Ley et al., 2008), this effect had to date been untested in Old World vultures. This demonstrates the resilience of vulture microbiota to captivity-related environmental and husbandry factors, whereby the vulture’s microbiome was most reflective of their carnivorous lifestyle.

As captive birds represent potential source populations for wild population recovery efforts, this resilience is of particular significance. However, our finding of a significant impact of one particular prey type (quail) requires further consideration as it represents a potentially important husbandry-associated influence on vulture microbiome. Quail may have acted as an inoculation source of Actinobacteria for Griffon vultures. This prey type has been shown to have a notably high abundance of Actinobacteria (Su et al., 2014) in contrast to the microbiome of rats (Li et al., 2017) and chickens (Oakley et al., 2014) that only includes Actinobacteria as a minor contributor. The lack of equivalent effect in Egyptian vultures may relate to our study design, which was not established to test this hypothesis and therefore our finding in Griffon vultures was not based on an experimental design established for the purpose of testing this. The relatively balanced split between fecal samples associated with quail and only one other prey species (rat) was fortunate, but the low number of days when the birds were fed other prey types may have impacted our ability to detect their influence. In contrast, Egyptian vultures were only fed quail on two occasions that could be temporally associated with samples used in analysis. Chicken was, however, associated with 12 samples but no influence of this prey type on fecal microbiome was detectable. Consideration is also required of the duration of prey type exposure. Our *post hoc* analysis of fecal samples evaluated according to the prey type consumed on the day prior to fecal voiding assumes that this ∼24 h period was sufficient to elicit an acute bacterial response. Although not commonly reported, there is evidence to demonstrate a rapid response to diet changes and that such acute bacterial changes are detectable within 24 h of feeding (Wu et al., 2011), thereby supporting our analytical approach.

An inoculating or modifying role for prey type has previously been shown in other birds of prey, including kites (Blanco, 2014), falcons and owls (Bangert et al., 1988) and New World vultures (Roggenbuck et al., 2014), whereby microorganisms identified in the hindgut of these raptors were considered to originate directly from the diet consumed. It is not possible to ascertain whether our findings represent an adaptation or inoculation effect of the luminal microbiome by prey type in our study. However, either mechanism is a particularly intriguing possibility in scavengers, given that these species are generally considered to have evolved efficient strategies to protect themselves against such inoculation. Concurrently, research in mice and humans has demonstrated an association between increased abundance of Actinobacteria and obesity and the consumption of high-fat diets (Clarke et al., 2012) such that the macronutrient content of prey offered in captivity is likely an important factor to consider. The implications of our findings in Griffon vulture remain to be elucidated but nonetheless represents an important anthropogenic influence, whereby free-ranging vultures (of any species) would not typically include large proportions of quail in their diet. Moreover, the increased abundance of *Nocardiaceae* should be interpreted with caution as these ubiquitous environmental bacteria are more likely to be transient passengers in the gastro-intestinal tract of vultures upon quail intake. However, they have been shown to act as opportunistic pathogens (including the genus *Rhodococcus*) in immunocompromised hosts (Barka et al., 2016). Elucidation of the functional importance of Actinobacteria may be facilitated once the microbiome of free-ranging individuals is characterized.

Whereas the implications of increased Actinobacteria abundance are as yet unknown, bacterial alignment with species-specific feeding strategies is still tangible here. These inter-specific differences should be considered when evaluating host–microbiota interactions, especially for animals intended for release to the wild. The notable lack of large ungulate carcass feeding for captive vultures (Gaengler and Clum, 2015) is at odds with their evolved dietary specialization, and reliance on smaller whole prey species may introduce important, but as yet unquantified, differences in bacterial communities. Whilst it is possible that a captive-to-wild bacterial composition transition may occur following release, e.g., most recently evidenced in Tasmanian devil’s (*Sarcophilus harrisii*) (Chong et al., 2019), this represents another acclimatization process, amongst a suite of other physiological and behavioral adaptations, incurred by released individuals. Since pre-release conditioning and training is already considered vital to post-release success, it would appear prudent that reintroduction programs include monitoring for (and mitigation against) captivity-induced microbiome alterations prior to release, alongside optimization of other health parameters, rather than leaving microbial adaptation to occur post-release. Given the importance of the microbiome to host health, the value of integrating microbiome knowledge into *ex situ* breeding program management is hereby emphasized.

Combined, these findings highlight the importance of species- and husbandry-specific drivers in shaping the gut bacterial community and cautions against inter-specific extrapolations. Captive breeding programs aimed at propagating vultures for release can be encouraged by the relative lack of influence that a more sanitized diet had on vulture gut microbiota; hygiene procedures implemented to protect human health do not appear to compromise vulture bacterial composition. The nutritional and behavioral implications of feeding such a sanitized diet were beyond the scope of this study but are nonetheless vital considerations when formulating captive vulture diets. The importance of incorporating microbial research in conservation practice is evident; most notably an understanding of species- and environment-specific effects should be considered fundamental to advancing knowledge necessary for implementing best practice in species recovery.

## Data Availability Statement

The 16S rRNA data sets generated in this study are made available and deposited in the NCBI Sequence Read Archive (SRA) under BioProject PRJNA621094 with BioSample accession numbers SAMN14501507 to SAMN14501558.

## Ethics Statement

The animal study was reviewed and approved by Nottingham Trent University’s School of Animal, Rural and Environmental Science Ethics Review Group (ARE76).

## Author Contributions

KW-T, SH, and GW-T conceived and designed the study with assistance from JB. KW-T and GW-T conducted the study, collected and prepared samples for laboratory analyses. AB completed all data analyses and interpretation, with input from SH and KW-T. KW-T and AB prepared and wrote the manuscript with input from all authors.

## Conflict of Interest

The authors declare that the research was conducted in the absence of any commercial or financial relationships that could be construed as a potential conflict of interest.
