# Supplementary material for: Integrating Gut Bacterial Diversity and Captive Husbandry to Optimize Vulture Conservation
Source: Front Microbiol. 2020 May 25;11:1025. doi: 10.3389/fmicb.2020.01025 (PMC7261900; doi:10.3389/fmicb.2020.01025)
Supplement: Supplementary file 1 [file Data_Sheet_1.docx]

Supplementary Material


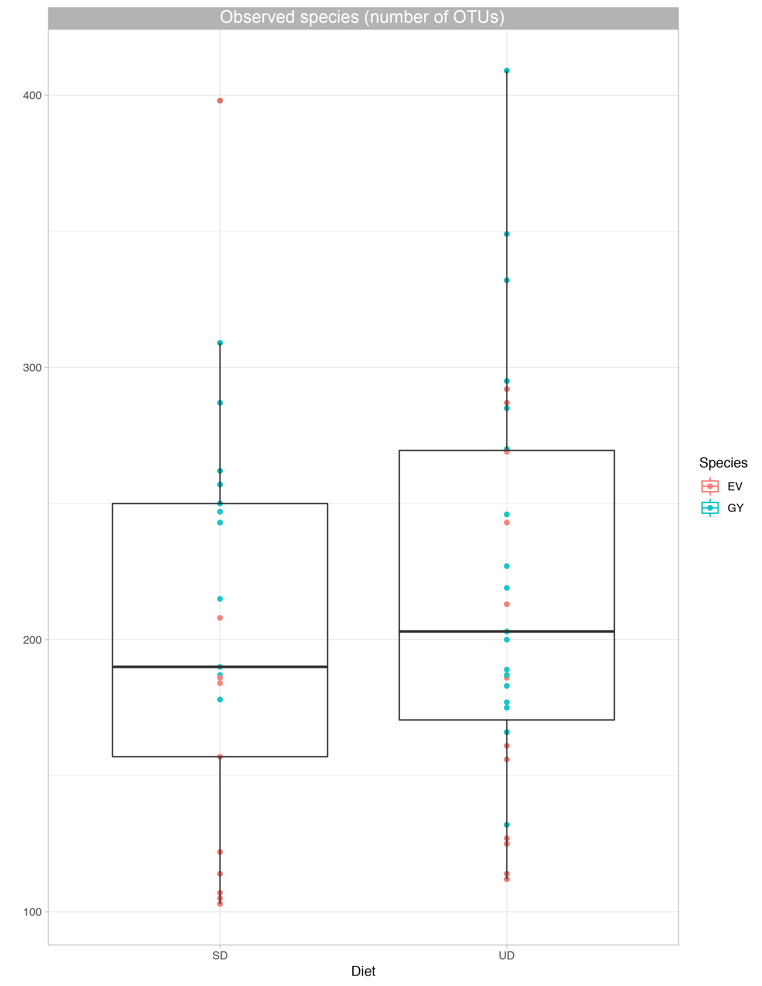


**Supplementary Figure 1**. **Variation in gut bacterial diversity between Old World vulture species fed either sanitized diet (SD) or unsanitized diet (UD).** Alpha diversity based on rarefied data, measured by observed species, plotted for 52 fecal samples of two Old World vulture species (EV = Egyptian vulture, 6 individuals, n = 22 samples; GY = Griffon vulture, 7 individuals, n= 30 samples). Vultures were fed either a sanitized diet (SD) consisting of skinned, de-gutted and washed rats, chicken and quail, or un-sanitized diet (UD) consisting of intact whole rats, chicken and quail. No significant differences were observed between diets (Wilcoxon, *P*>0.05).


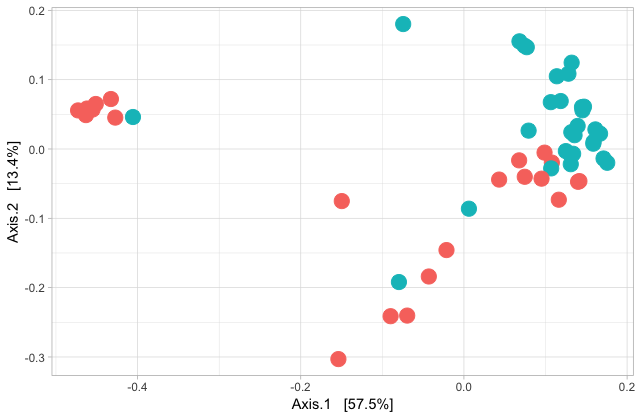


**Supplementary Figure 2**. **Egyptian and Griffon vultures exhibit different bacterial communities.** Beta diversity; principal coordinate analysis visualizing the clustering of bacterial communities of 52 fecal samples from Egyptian (6 individuals, n= 22; red) and Griffon vultures (7 individuals, n= 30; blue) based on weighted UniFrac dissimilarity matrix.

**
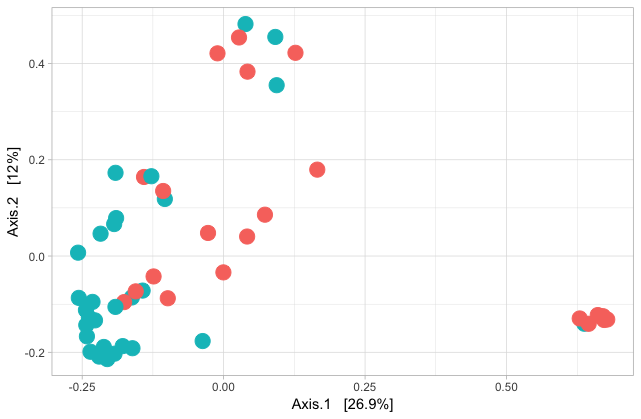
**

**Supplementary Figure 3**. **Egyptian and Griffon vultures exhibit different bacterial communities.** Beta diversity; principal coordinate analysis visualizing the clustering of bacterial communities of 52 fecal samples from Egyptian (6 individuals, n= 22; red) and Griffon vultures (7 individuals, n= 30; blue) based on Bray-Curtis distance.


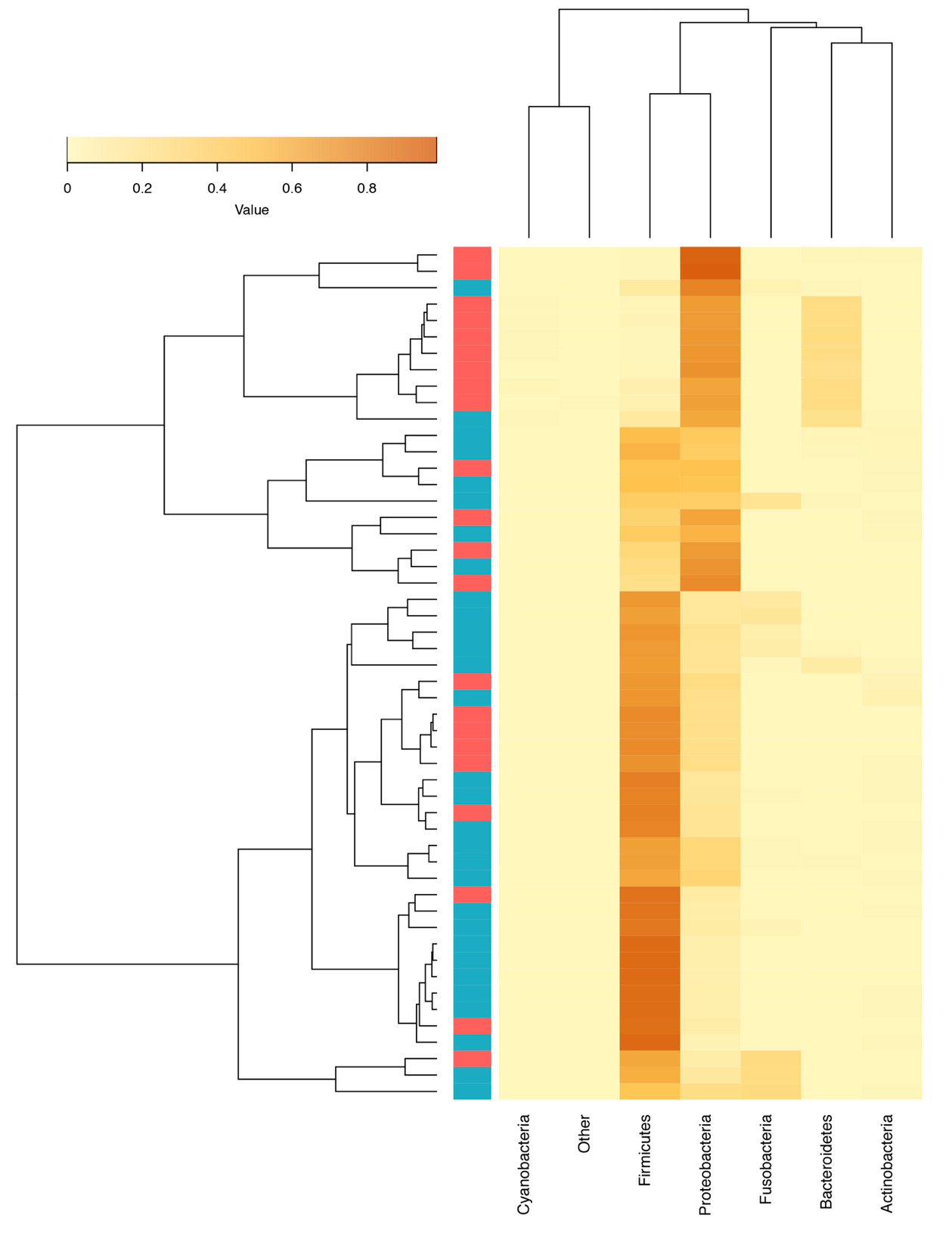


**Supplementary Figure 4**. Relative abundance of phyla in fecal samples of Egyptian and Griffon vultures. Heatmap showing the relative abundance of phyla in 52 fecal samples from Egyptian (6 individuals, n= 22; red) and Griffon vultures (7 individuals, n= 30; blue). Hierarchical cluster analysis was based on the Bray-Curtis distance with average-linkage method. The color code indicates the relative abundance of OTUs per sample.
